# Comparison of Environmental and Culture-Derived Bacterial Communities through 16S Metabarcoding: A Powerful Tool to Assess Media Selectivity and Detect Rare Taxa

**DOI:** 10.3390/microorganisms8081129

**Published:** 2020-07-27

**Authors:** Jacques Pédron, Léa Guyon, Amandine Lecomte, Lydie Blottière, Charlotte Chandeysson, Emma Rochelle-Newall, Xavier Raynaud, Odile Berge, Marie-Anne Barny

**Affiliations:** 1 INRAE, IRD, Institute of Ecology and Environmental Sciences-Paris, Sorbonne Université, IEES-Paris, F-75231 Paris, France; jacques.pedron@upmc.fr (J.P.); lguyon2@clipper.ens.fr (L.G.); lecomteamandine1@gmail.com (A.L.); lydie_blottiere@yahoo.fr (L.B.); emma.rochelle-newall@ird.fr (E.R.-N.); xavier.raynaud@upmc.fr (X.R.); 2 INRAE, Pathologie Végétale, F-84143 Montfavet, France; charlotte.chandeysson@inrae.fr (C.C.); odile.berge@inrae.fr (O.B.)

**Keywords:** 16S barcoding, *Pectobacterium*, *Pseudomonas*, river, bacterial communities, medium selectivity, cultivation, microbial ecology

## Abstract

To compare environmental and culture-derived microbial communities, we performed 16S metabarcoding of uncultured samples and their culture-derived bacterial lawns. Microbial communities were obtained from freshwater river samples representative of an anthropization gradient along a river stream. Their culture-derived bacterial lawns were obtained by growing aliquots of the samples on a broad range medium and on two different semi-selective media. The V3–V4 16S rRNA region was amplified and sequenced. The bacterial diversity of water samples decreased from the upper to lower stream sampling sites and, as expected, these differences were mostly suppressed by the culture step. Overall, the diversity of cultured-derived bacterial communities reflected selectivity of each tested medium. Comparison of treatments indicated that the culture selected both detected and rare undetected environmental species. Accurate detection of rare environmental bacteria of the *Pectobacterium* genus by 16S metabarcoding of the culture lawn was demonstrated. Interestingly, for abundant taxa, such as those of the *Pseudomonas* genus, the culture/environment ratio varied between sampled sites, indicating the difficulty of comparing cultured-derived taxa abundance between environmental sites. Finally, our study also highlighted media specificity and complementarity: bacterial communities grown on the two selective media, while selecting a small set of specific species, were mostly a subset of the bacterial community observed on the broad range medium.

## 1. Introduction

Advances in culture independent methodologies have revolutionized our understanding of microbial diversity. The 16S rRNA gene is currently the most commonly used target in the analysis of bacterial diversity [1]. When combined with high throughput sequencing (HTS), the 16S rRNA gene offers the opportunity to easily, rapidly, and efficiently describe a bacterial community in a given environment [2]. The number of bacterial species identified through 16S rRNA gene sequence analysis is far greater than the number of bacterial species identified on a given culture medium. It is estimated that approximately 80% of bacteria detected with molecular tools are uncultured [3]. As a result, a large number of bacterial groups are only known as operational taxonomic units (OTUs), and their ecological role and physiology are difficult to study. However, 16S metabarcoding approaches, while giving a robust and broad view of the bacterial community, are not adapted to dealing with the rare biosphere that represents an enormous complement of abundant taxa, very important for the functioning of ecosystems [4,5,6]. In addition, 16S rRNA-based taxonomy is often not able to discriminate between closely related innocuous and virulent organisms. Furthermore, culturing microorganisms also opens the possibility of studying the ecological role and physiology of cultured bacteria including their potential virulence as suspected pathogens. To complete 16S-driven approaches of microbial diversity, culture-based approaches are thus necessary.

Consequently, while many environmental studies focus on large descriptions of microbial diversity through HTS approaches, other environmental studies rely on culturing approaches to estimate the abundance of a given culturable taxa in the environment. Both approaches are usually performed independently, and this does not allow direct comparison of the benefits and limits of both methods. For example, it remains unclear, when a specific taxon in the environment is detected through the culture-based approach, if this taxon belongs to a rare or an abundant taxon in the environment. In addition, as cultured-based approaches select only a subset of culturable bacteria, it remains unclear to what extent culture driven enrichments could be compared between different environmental samples. Furthermore, as different media have different selectivity, it is also difficult to compare the abundance of different culturable bacteria selected on different media in the same environmental sample.

We present a fast and accurate method, based on direct 16S metabarcoding of a bacterial lawn without isolation of the grown bacterial colonies, to rapidly evaluate and compare culture media selectivity with uncultured environmental samples. The three studied media were TSA 10%, a commonly used, broad range medium [7]; KBC, a semi-selective medium used to detect bacteria of the *Pseudomonas syringae* group [8,9]; and CVP, a semi-selective medium often used to detect soft rot pectinolytic bacteria (SRP) of the *Pectobacterium* and *Dickeya* genera [10]. To test our protocol, river surface water, sampled from different sites along an anthropic gradient in the Durance river stream, was spread on media plates. The diversity of bacterial communities grown on each medium was compared with the diversity observed in the uncultured river water samples as well as with the one measured on the two other tested media. 16S metabarcoding allowed rapid comparison of all the treatments.

## 2. Materials and Methods 

### 2.1. Sampling, Culture on Selective Media, and DNA Extraction

**Figure 1:**
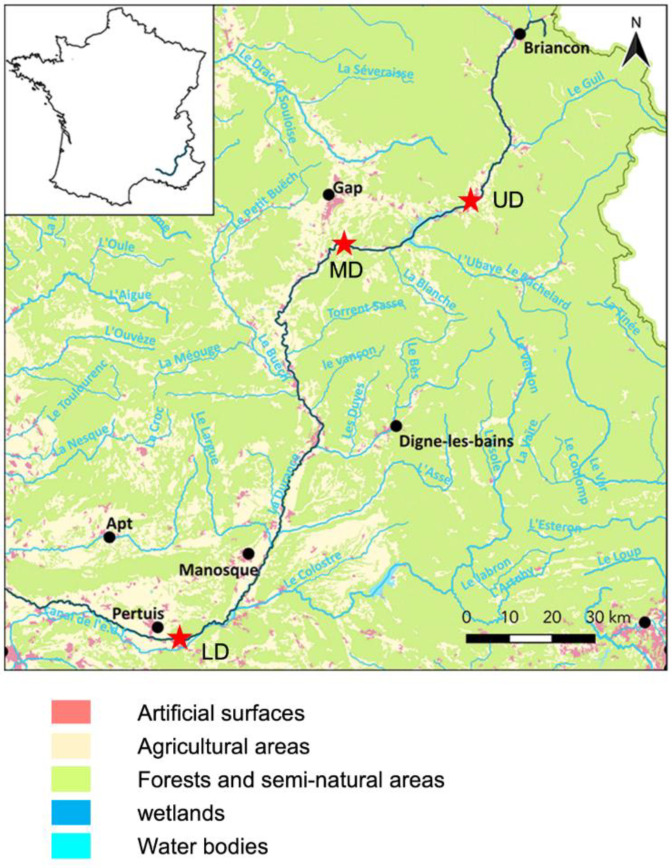
Geographic distribution of sampling sites along the Durance River. The Durance River is located in the southeast of France. The spring is located in a pristine alpine area at an altitude of 2390 m, while the confluence with the Rhône River is located at an altitude of 10 m surrounded by agricultural land. The red stars indicate the location of the three sampling sites. UD: Upper Durance, MD: Middle Durance, LD: Lower Durance.

Sampling of surface water was performed at three sites, along the Durance River in the southeast of France (Figure 1). The Upper Durance sampling site (UD) is located at the bottom of the alpine part of the river. The Middle Durance sampling site (MD) is located in the upper part of agricultural land dominated by apple and pear production. The Lower Durance sampling site (LD) is located in the lower part of agricultural land with intensive production of fruits, cereals, and vegetables. At each site, surface river water was sampled (3 L) on the 13th of May 2016. Sampling sites are described in Figure 1.

**Figure 2:**
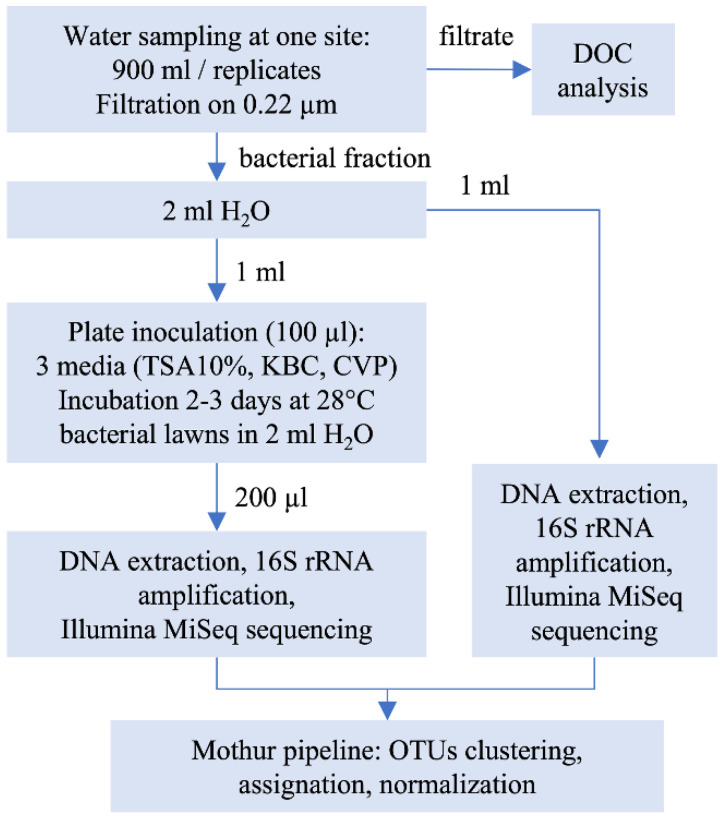
Overview of the experimental procedure.

In situ temperature and electrical conductivity were measured using a Multi Probe System (YSI 556 MPS) and water turbidity was measured using a EUTECH Instruments turbidity meter (Table 1). Samples were maintained in a cool box before treatment that occurred within 24 h. Samples were split in 3 replicates of 900 mL that were individually filtered through 0.22 µm cellulose acetate filters (Sartorius). Experimental procedures are summarized in Figure 2. The fraction retained on the filter was suspended in 2 mL of sterile distilled water and 1 mL was frozen at −20 °C until DNA extraction was performed (“environmental” sample). The remaining 1 mL was used to inoculate plates (100 µL per plate) of the following media: TSA 10% [7], KBC [8,9], or CVP [10]. The plates were incubated at 28 °C for 2 (CVP and TSA 10%) or 3 days (KBC). Two mL of sterile water was added to the bacterial lawn, and bacteria were scratched out from the lawn and suspended in the water with a spreader. An aliquot of 200 µL of the obtained suspension was used for DNA extraction (“culturable” samples). All DNA extractions were performed using the Wizard^®^ Genomic DNA Purification Kit (Promega, Madison, WI, USA).

To estimate the culturable bacterial population grown on plates, serial dilutions of the sampled river water were performed (from 10^−1^ to 10^−5^), plated on each media and incubated at 28 °C. The bacterial counts obtained were used to estimate the number of bacteria grown on the 10^0^ TSA 10%, KBC, and CVP plates used for DNA extraction. To estimate which soft rot *Pectobacteriaceae* (SRP) were recovered during the experiment, a second 10^0^ CVP plate was used. The pit-forming bacteria on this plate were isolated and the genus of each individual bacterium (*Dickeya* or *Pectobacterium*) was determined following amplification and sequencing of the *gap*A housekeeping gene, as previously described [11]. The number of bacteria from the *P. syringae* group (as delineated by Mulet et al. [12]) in samples was estimated by plating 10^−1^ sample dilutions on the KBC medium. Putative *P. syringae* colonies were enumerated, and their affiliation to the *P. syringae* group was checked using the specific *P. syringae* PCR primers for a representative set of 30 isolates/sample. *P. syringae* abundances were then calculated [13,14].

### 2.2. Water Chemical Analysis

The filtrate solution of river water samples was used to determine the dissolved organic carbon (DOC) concentration, as described in [15]. Briefly, two 30 mL samples were transferred to pre-cleaned glass vials; the samples were then acidified with 35 µL 85% H_3_PO_4_ and sealed with a Teflon lined cap. Samples for measurement of the optical properties of the colored fraction of the bulk DOC pool, colored or chromophoric dissolved organic matter (CDOM), were collected in 90 mL pre-cleaned amber-colored glass bottles and sealed with a Teflon lined cap. Samples were stored frozen until measurement. Absorption was measured with a spectrophotometer (Analytica.Jena Specord 205 UV-VIS) from 200–750 nm using a 1 cm quartz cell and Milli-Q water as the blank [11]. The spectral slope ratio (S_r_) was determined from the ratio of the spectral slope of the absorption coefficient over the ranges 275–295 nm and 350–400 nm [16]. S_r_ is considered to be a proxy for apparent molecular weight, where higher values indicate lower molecular weight dissolved organic matter (DOM), lower aromaticity, and potentially higher DOM bioavailability [17]. Fluorescence was determined with a Turner Trilogy fluorometer with the CDOM insert. Values were expressed as relative fluorescence units (RFU) [11].

### 2.3. DNA Amplification and Sequencing

The 16S rRNA gene V3–V4 variable region was amplified using PCR primers 341F (CCTACGGGNGGCWGCAG) and 805R (GGACTACHVGGGTWTCTAAT), with a barcode on the forward primer. PCR using the HotStarTaq Plus Master Mix Kit (Qiagen, Hilden, Germany) was performed under the following conditions: 94 °C for 3 min, followed by 28 cycles of 94 °C for 30 s, 53 °C for 40 s, and 72 °C for 1 min, after which a final elongation step at 72 °C for 5 min was performed. Amplified PCR products were checked in 2% agarose gel to determine the success of amplification and the relative intensity of bands. Multiple samples were pooled together in equal proportions based on their molecular weight and DNA concentrations. Pooled samples were purified using calibrated Ampure XP beads and used to prepare the Illumina DNA library.

Sequencing was performed at MR DNA (www.mrdnalab.com, Shallowater, TX, USA) on a MiSeq sequencer following the manufacturer’s guidelines. Sequence data were processed using the MR DNA analysis pipeline (MR DNA, Shallowater, TX, USA). In summary, sequences were joined, depleted of barcodes, and sequences < 150 bp or with ambiguous base calls were removed.

### 2.4. Clustering, Alignment, and Phylogenetic Analysis of 16S rRNA Gene Fragments

The whole sequence set was analyzed using the Mothur pipeline v.1.39.5, dedicated to the study of microbial communities [18], according to the standard operating procedure (www.mothur.org/wiki/MiSeq_SOP) [19]. Briefly, sequences were aligned to the SILVA SSU non-redundant database (v.123, www.arb-silva.de), and sequences deviating from consensus at the start and end positions and chimera sequences were removed. Sequences were then clustered in OTUs (operational taxonomic units) using a cut-off of 97% identity. OTUs were taxonomically classified at the genera and phylum level with the Ribosomal Database Project (RDP) Bayesian classifier [20].

### 2.5. Data Analysis

Several ecological indexes were calculated to characterize the bacterial community present following each treatment. To characterize the α-diversity, that is, the diversity present in each sample, we used 3 different indexes. The count of all the different OTUs characterized the richness of the diversity observed in each sample. This index indicates how many different taxa are present in the sample. The Pielou Index compares the abundance level of each taxon present in the sample. It allows deciphering if the taxa are evenly distributed or if some dominate the others. The Shannon Index is a classical ecological index, often referred to as the α-Diversity Index, which takes into account both the diversity and the evenness of the taxa present in the sample. To characterize the dissimilarities between samples (β-diversity), the Bray–Curtis Index was calculated. As this index mixes the differences due to species losses and species turnover, we also partitioned this index to understand both components of dissimilarity.

All analyses were performed after removal of singleton sequences. Normalization of the sample size (number of sequences per sample) was achieved by randomly resampling the same number of reads for each sample based on the smallest sample size (*n* = 31,087, Table S1). Rarefaction curves, Shannon’s Index (α-diversity), and Pielou’s Index (evenness) were calculated with the PAleontological STatistics (PAST) software v.3.20 [21]. The mean and standard deviation of the 3 replicates were calculated for each sample. Statistical significance was assumed at the 5% level (independent two-sample student *t*-test, unequal variance, two-sided *p*-value < 0.05). Tables S2–S4 provide the *p*-values of all performed statistical tests.

The bacterial community structure was analyzed with the MEGA v.5.11.3 software after normalization (ab.inf.uni-tuebingen.de/software/megan/; Huson et al., 2007). A Bray–Curtis [22] dissimilarity matrix was calculated with the statistical software XLSTAT v.19.6 (Addinsoft, USA, www.xlstat.com) from the genera abundance matrix. The dissimilarity matrix was visualized by a two-dimensional multidimensional scaling (MDS) using XLSTAT. The total β-diversity was partitioned in terms of turnover (genera replacement, β_sim_) and nestedness (genera loss or gain, β_nes_) between samples using the betapart R package [23].

## 3. Results

### 3.1. Sampling Sites, Water Parameters, and Culture Conditions

Sampling of surface water was performed at three sites along the Durance River chosen for their differential altitudes and surrounding land uses and, respectively, called the UD (Upper Durance), MD (Medium Durance), and LD (Lower Durance) (Figure 1). As expected, the water temperature was higher for the LD sampled water as compared to the UD and MD sampled waters. Similarly, turbidity was higher for the UD sampled water (Table 1). Dissolved organic carbon (DOC) concentrations were comparable at the three sites. However, a strong difference in fluorescence and carbon normalized fluorescence was observed between the three sites, indicating differences in the quality of the carbon present. A similar pattern was observed for carbon derived organic matter (CDOM) absorption at 355 nm and for the spectra slope S_r_ (Figure S1). These results suggest that DOM in the MD site could be less bioavailable than in the UD and LD sites.

The number of colony-forming units (CFUs) observed on the semi-selective media CVP and KBC were of the same order of magnitude while, as expected, a much larger number of CFUs could be detected on the broad range TSA 10% medium (Table 1). Bacteria belonging to the *P. syringae* group were estimated on KBC plates, and a net decrease of *P. syringae* detection was observed from the UD to LD sites (Table 1). The colonies forming pits, a characteristic of SRP bacteria grown on the CVP medium, were also counted. Pits forming bacteria were only visually detected in the LD site (Table 1). Their subsequent isolation and molecular characterization showed that all the pits forming bacteria detected belonged to the *Pectobacterium* genus.

### 3.2. General 16S Metabarcoding Data and α-Diversity

A total of 3,116,952 sequences were obtained after Illumina sequencing. The assigned sequenced reads of each sample ranged from 31,087 to 77,427, from which 249 to 4141 OTUs with more than one read were described depending on samples, treatments, or replicates (mean per replicates are given in Table 2). Rarefaction curves (Figure S2) nearly reached a plateau while Good’s coverage estimates (Table 2) showed that less than 1% of reads were from OTUs that appeared only once in the analyzed samples. This indicates that the sample coverage was correct and could be used to describe the diversity. 

As expected, direct 16S metabarcoding of environmental samples showed that richness (OTU numbers), α-diversity (Shannon Index), and evenness (Pielou Index) significantly decreased from the upstream to the downstream parts of the river (Table 2 and Table S2). However, these differences between sites almost disappeared when analyzing the culturable samples (Table 2 and Table S2). Indeed, whatever the studied site (UD, MD, or LD), the richness, α-diversity, and evenness indexes were generally not statistically different on a given medium (Table S2). However, a few exceptions were observed: α-diversity and evenness were found to be statistically different between the MD and LD sites on the TSA 10% medium (*p*-value of 0.008 and 0.01, respectively, Table S2). Similarly, richness on the KBC medium was statistically different between the UD and MD sites and between the MD and LD sites (*p*-value of 0.015 and 0.025, respectively, Table S2).

Comparison between media indicated that, as expected, richness, α-diversity, and evenness were generally higher on the broad range TSA 10% medium than on the semi-selective KBC or CVP media (Table 1 and Table S3). In contrast, richness, α-diversity, and evenness were mostly comparable between the CVP and KBC media, indicating a comparable selective force of these two media (Table 1 and Table S3).

**Figure 3:**
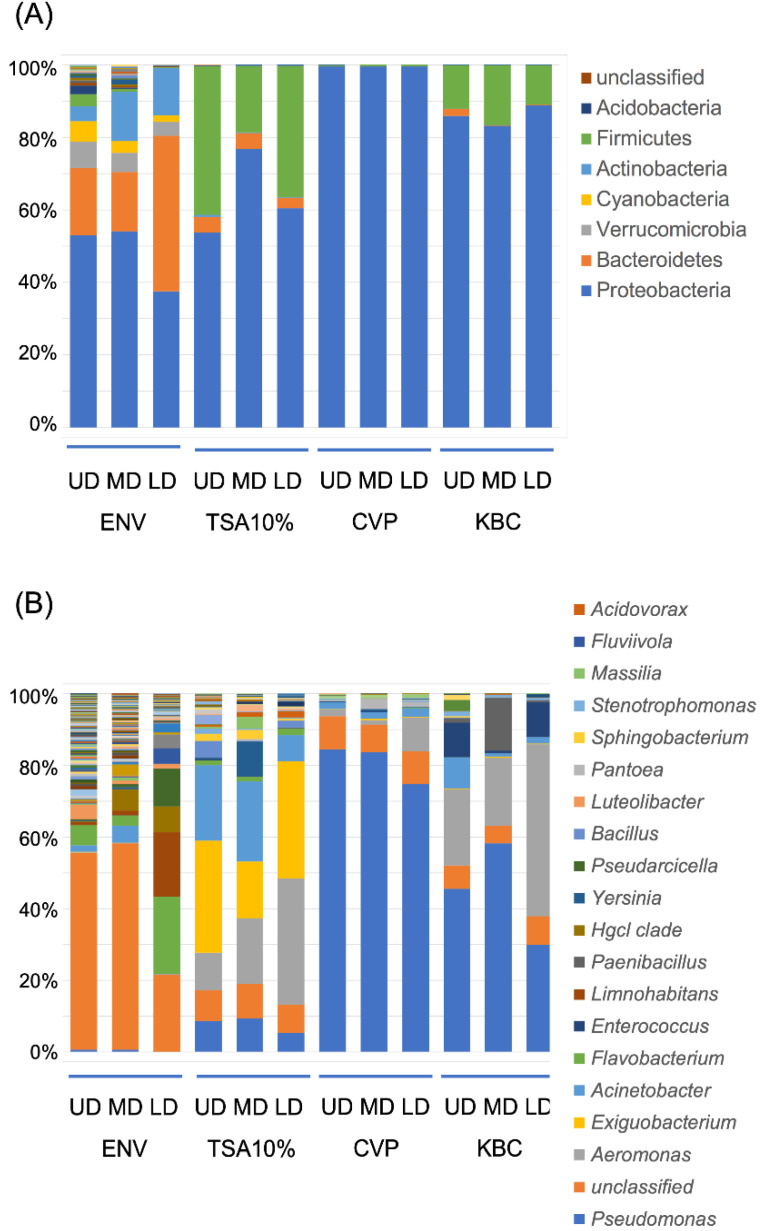
Bacterial structure communities at the phylum (**A**) and genus (**B**) level. ENV: environmental sample, TSA: TSA 10% cultured sample, CVP: CVP cultured sample, KBC: KBC cultured sample, UD: Upper Durance, MD: Middle Durance, LD: Lower Durance. Legends for phyla (**A**) and genera (**B**) were restricted to those exceeding 1% of total reads. For each condition, each replicate was normalized (*n* = 31,087) and the three replicates were averaged.

A qualitative assessment on the recovered community was performed following assignment of the observed OTUs to phyla and genera (Table 2 and Figure 3). Among the three water environmental samples, the OTUs were affiliated to up to 42 phyla, of which six (Proteobacteria, Bacteroidetes, Verrucomicrobia, Cyanobacteria, Actinobacteria, and Firmicutes) dominated the environmental bacterial communities, regardless of the studied site (Figure 3a). Nevertheless, the abundance of these dominating phyla varied between sites. For example, one could observe a large increase of Bacteroidetes at the LD site compared to the UD and MD (Figure 3a). These changes in bacterial composition were also highlighted at the genus level. The numbers of observed genera significantly decreased from the UD to LD sites (Table 2 and Table S2). While many genera were no longer dominant at the LD site, others such as *Flavobacterium* and *Limnohabitans* became dominant (Figure 3b).

As expected, a drastic reduction of phyla could be observed following culture step, with each medium selecting a comparable number of phyla (Table 2 and Table S3). The phylum Proteobacteria accounted for more than 50%, 80%, and 99% of the total OTU reads on the TSA 10%, KBC, and CVP media, respectively (Figure 3a). Several phyla initially abundant in river water such as Actinobacteria, Verrucomicrobia, and Cyanobacteria were no longer abundant while Firmicutes and, to a lesser extent, Bacteroidetes phyla were still abundantly detected on the TSA 10% and KBC media but not on the CVP medium (Figure 3a).

Differences between media were even more enhanced at the genera level. More genera were identified on the TSA 10% medium compared to the CVP or KBC media, while no significant differences were observed between the two latter (Table 2 and Table S3). The four major genera observed on the TSA 10% culture were *Aeromonas*, *Acinetobacter, Exiguobacterium,* and *Pseudomonas,* and their relative proportion changed between study site (Figure 3b). *Pseudomonas* and *Aeromonas* genera were abundant on the KBC and TSA 10% media, while *Flavobacterium* and *Enteroccocus* genera were not selected on the TSA 10% but were found on the KBC medium. Cultures on the CVP medium mostly selected the *Pseudomonas* genus (Figure 3b).

### 3.3. Comparison between Environmental and Culture Treatments

**Figure 4:**
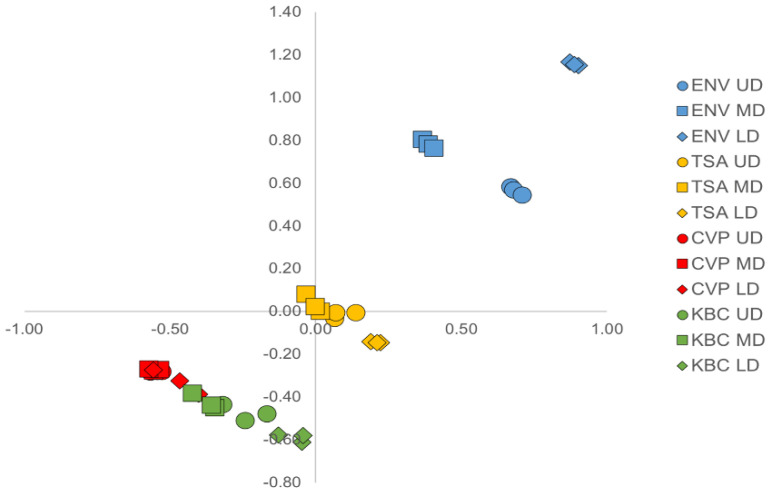
Multidimensional scaling visualization of the Bray–Curtis dissimilarity sample matrix. For each sample, each of the three replicates is plotted. Kruskal stress: 0.037. ENV: direct 16S metabarcoding of sampled water (blue), TSA: TSA 10% cultured samples (yellow), CVP: CVP cultured sample (red), KB: KB cultured sample (green). UD: Upper Durance (circle), MD: Middle Durance (square), LD: Lower Durance (diamond).

To compare the different treatments, we calculated the Bray–Curtis dissimilarities between sites, treatments, and replicates. Visualization of the dissimilarity between sites, treatments, and replicates by two-dimensional multidimensional scaling analysis (MDS) clearly separated the environmental samples (blue), the TSA 10% culturable samples (yellow), and the KBC/CVP culturable samples (green /red), which were grouped together (Figure 4 and Table S4). The three sites, UD (circle), MD (squares), and LD (diamonds), were clearly separated for the three environmental water samples on both dimensions of the MDS projection, and to a lesser extent for the TSA 10% or KBC media. On the other hand, the three sampling sites were mostly regrouped following growth on the CVP medium.

**Figure 5:**
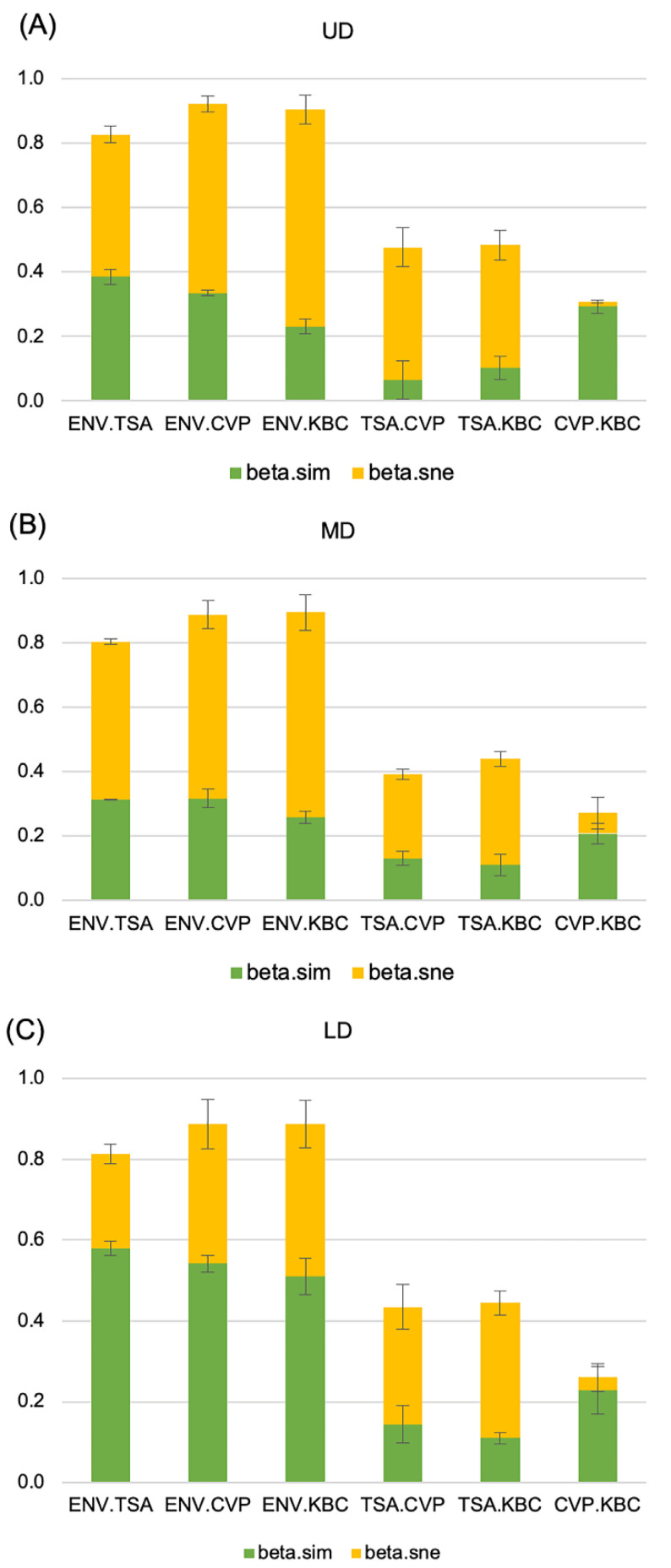
β-diversity partitioning between treatments. Each bar on the histogram represents the β-diversity between two different treatments. The two components of β-diversity, turnover (β.sim) and nestedness (β.sne), are respectively represented in green and yellow. Errors bars represent the standard deviation between the three replicates. ENV: direct 16S metabarcoding of sampled water, TSA 10%, CVP, or KBC: 16S metabarcoding after respective growth on TSA 10%, CVP, or KBC. UD: Upper Durance, MD: Middle Durance, LD: Lower Durance.

The dissimilarity between sites, which is referred to as β-diversity, could reflect two different ecological situations. First, it could reflect the loss of species from one site compared to the other. In that case, the species present at one site are nested inside a larger set of species present at the other site. Alternatively, each site could be different because they host none of the species present at the other site. In that second case, there is a complete turnover of species between the two sites. In practice, both events occur at the same time and the Bray–Curtis Index takes into account both components of dissimilarity. To understand which type of difference occurred between treatments, we partitioned the Bray–Curtis dissimilarity into its two components: turnover (β-sim), which reflects differences in composition caused by the replacement of some species by others (turnover), and nestedness (β-ness), which accounts for differences in species composition caused by species losses that can be observed when species of one site are a subset of species of another site displaying higher richness [24,25]. Between the environmental and cultured samples, a high dissimilarity was observed, whatever the culture conditions tested, with both turnover and nestedness accounting for the observed dissimilarity (Figure 5). However, the nestedness component was more important for the UD and MD sites, while a larger impact of turnover was observed for the LD site. Comparison between media showed that the dissimilarities between the TSA 10% culture medium and the KBC or CVP culture media were mostly due to nestedness, highlighting that the semi-selective KBC and CVP media mostly selected a subset of species that were also selected on the broad range TSA 10% medium (Figure 5). Finally, the low dissimilarity between the CVP and KBC culture treatments was mostly due to turnover replacement, accounting for the differential selectivity of both media for a small but different set of culturable species (Figure 5).

**Figure 6:**
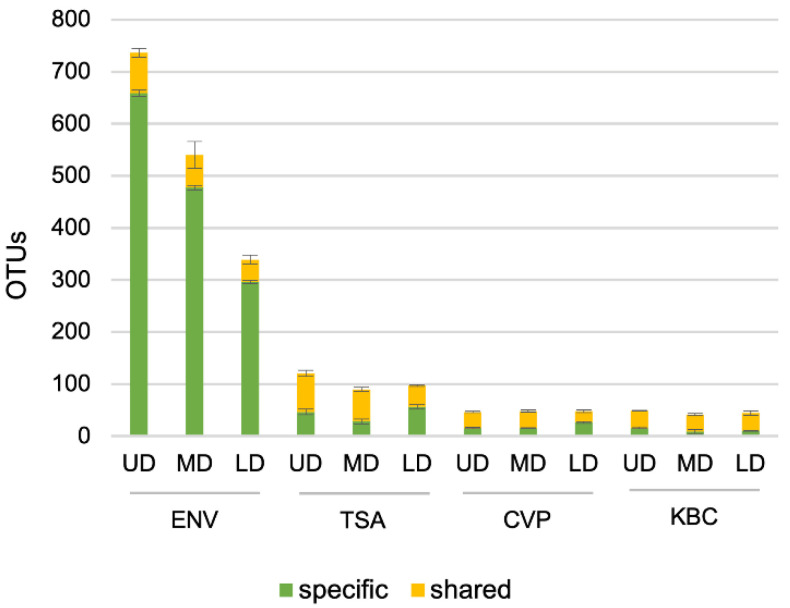
Analysis of specific/shared operational taxonomic units (OTUs) between cultured and environmental samples. For environmental samples, bars represent specific and shared OTUs between environmental and all cultured samples. For cultured samples, bars represent specific and shared OTUs between each cultured medium and environmental samples. Error bars represent the standard deviation between the three replicates.

To confirm and extend the above analysis, we counted the number of OTUs specific for each treatment and the number of OTUs shared between environmental and cultured treatments at each site (Figure 6 and Table S5). The number of OTUs specific to the environmental samples was high, indicating, as expected, that the three studied media selected only a very small part of the diversity observed in the environmental samples. However, culture-specific OTUs not detected in the environmental samples were observed on cultured samples. Environment-specific OTUs decreased from the UD to LD, whereas the total number of OTUs observed on a specific medium was mostly stable between sites, whatever the observed environmental diversity.

### 3.4. Culture Detection of Abundant Environmental OTUs: Focus on the Pseudomonas Genus

Among the three environmental samples, OTU reads assigned to the *Pseudomonas* genus accounted for 0.49%, 0.49%, and 0.09% of the normalized OTU reads studied in the UD, MD, and LD sites, respectively (Table 3). All media strongly selected the *Pseudomonas* genus, and OTUs assigned to this genus accounted for at least 5.39% (TSA 10%—LD site) and at most 84.41% (CVP—UD site) of the total reads per treatment (Table 3). Calculation of the culture enrichment ratio showed that, whatever the tested medium, the culture enrichment ratio media:environment varied between sites. This ratio was larger when the abundance of the *Pseudomonas* genus in the initial environmental sample tested was lower.

### 3.5. Culture Detection of Rare OTUs: Focus on the Pectobacterium Genus

In contrast to what was observed with *Pseudomonas,* no read was assigned to OTUs belonging to the *Dickeya* or *Pectobacterium* genera through direct 16S barcoding of the three environmental samples (Table 4), indicating that both *Pectobacterium* and *Dickeya* genera belong to the rare biosphere in river water. Consequently, we could not calculate a culture enrichment ratio. However, 51 colonies forming pits were observed on the three CVP plates at the LD site, accounting for 0.48% of the total CFUs counted on these CVP plates (Table 4). Accordingly, a large number of reads (928) corresponding to *Pectobacterium* OTUs were observed following 16S barcoding of these CVP cultured plates, accounting for 0.99% of the total reads (Table 4). This indicated that 16S barcoding of the culture medium reflected the colonies’ growth, even when only a few colonies of a given genus were growing on the lawn. Interestingly, 16 reads were also observed following 16S barcoding of the CVP cultured plates of water sampled at the UD site, suggesting that a small pit-forming colony was present on one of the three analyzed plates but remained undetected visually (Table 4). Alternatively, this small number of reads could have arose from well-to-well contamination between samples during sample treatments, as already described [26].

## 4. Discussion

To rapidly compare environmental and culture-derived bacterial communities, we performed 16S metabarcoding of environmental and culture-derived samples. The three environmental studied sampling sites were chosen to harbor variable bacterial communities representative of an agricultural and anthropogenic gradient along the Durance River. As expected, direct 16S metabarcoding analysis of environmental river water samples showed a change in the structuration of bacterial communities, with a decrease of richness, diversity, and evenness from the upstream sampling site to the downstream sampling site. This suggests a shift from a generalist microbial community present in oligotrophic water at the highest sampling point to a more specialized microbial community further down, as previously observed in other river streams [27,28,29]. This change could be due in part to differences we observed in dissolved organic carbon quantity and quality between the three samples, as it has previously been shown that DOC strongly structures bacterial communities in aquatic environments [30,31,32].

16S metabarcoding of culture-grown bacteria, as expected, largely erased the diversity differences observed between the three environmental freshwater samples. However, on the TSA 10% culture medium, the Shannon and Pielou indexes were still found to be significantly different between the MD and LD sites, and richness on the KBC medium was also statistically different between the UD and MD. As well, the sampling site could still be distinguished with both cultured samples though MDS analysis of β-diversity, further indicating that both media still catch some of the differences between sampling sites.

16S barcoding analysis allowed straightforward comparison of environmental and culture-derived samples. β-diversity partitioning between environmental and cultured-derived samples showed that turnover and nestedness accounted almost equally for the total diversity changes between the environmental and cultured-derived samples, indicating that culture-driven enrichment applied to both detected and undetected environmental species. Interestingly, the relative proportion of nestedness and turnover components varied between sites, with a larger impact of turnover observed for the lowest diversity LD site (Figure 5). More importantly, as exemplified with the *Pseudomonas* genus, the culture enrichment ratio media:environment varied between sites, culture enrichment being larger when *Pseudomonas* abundance was lower in the initial environmental sample (Table 4). This indicates that the culture step selected culturable species, whatever their abundance in the initial environmental sample. Therefore, the variation in the enrichment ratio made comparison of a given taxa abundance between environmental sites problematic through culture analysis.

The culture step revealed many bacterial OTUs that were not detected through 16S metabarcoding of the environmental water samples (see Table S1 for detail). Since both environmental and culture-derived samples were analyzed through the same 16S-barcoding procedures with the same primers, one could eliminate PCR biases between the analyzed samples and, therefore, these culture-specific OTUs most probably represent genera that were rare in the initial environmental water sample. Interestingly, the coherence between the proportion of *Pectobacterium* sp. colonies visually detected on the CVP plates and the proportion of 16S reads assigned to OTUs of the *Pectobacterium* genus indicates that rare bacterial colonies were correctly assessed through 16S metabarcoding of cultured bacterial lawns. Therefore, to identify a rare species of interest, combining a culture step on a semi-selective medium and metabarcoding analysis is powerful and can reduce the costs of detection. 16S metabarcoding appears especially useful if the culture medium does not allow discriminating visually the few rare colonies of interest from other colonies growing on the semi-selective medium as it reduces the labor-intensive characterization of individual colonies. Possibly, for such an analysis, the choice of primers could be adapted to reach the species level depending on the species of interest. For example, primers designed on the *gap*A housekeeping gene [14] discriminate the various SRP species, while the *cts* housekeeping gene proved powerful for discriminating the various clades within the *P. syringae* group [32].

Analysis of cultured samples through 16S metabarcoding allowed to rapidly compare the specificity of each tested medium. The selective force of each medium could be inferred from the calculated richness, diversity, and evenness indexes (Table 2). As expected, the broad range TSA 10% medium had the lowest selective force, while the semi-selective CVP and KBC media appeared to have a comparable selective force. Assessment of media selectivity appears useful if one wants to compare the environmental abundance at a given environmental site of different species that could be isolated on different media. For example, the KBC media is regularly used to detect phytopathogenic bacteria of the *P. syringae* group [8,9], while CVP detect phytopathogenic bacteria of the genera *Dickeya* and *Pectobacterium* [10,33,34]. Since these two media have a comparable selective force, the abundance of *P. syringae* and *Dickeya/Pectobacterium* at each site could be compared. This led us to conclude that *P. syringae* and *Pectobacterium/Dickeya* populations had a strong differential abundance at the UD and MD sites but were comparable at the LD site (Table 1). Analysis of β-diversity between the culture media also allowed rapid comparison of media specificity. Indeed, the higher the β-diversity turnover between two media, the more each medium selects a different set of species. Therefore, if one wants to cultivate the largest diversity of bacteria from a given environmental sample, it is important to choose a set of media with high β-diversity turnover. This could be rapidly analyzed through 16S metabarcoding of cultured samples.

16S metabarcoding also allowed a rapid qualitative assessment of the bacteria grown on the media. For example, Gram-positive bacteria belonging to the Firmicutes phylum were still abundantly detected on the KBC medium, although cephalexine was used to counter-select Gram-positive bacteria on this medium. In contrast, crystal violet, used for the same purpose in the CVP medium, appeared much more efficient in counter-selecting Gram-positive bacteria since Gram-positive bacteria were not detected. Our analysis also revealed an unexpected feature: the CVP culture medium, set up to isolate the genera *Pectobacterium* and *Dickeya,* appeared more selective for the *Pseudomonas* genus than the KBC culture medium, used to detect bacteria of the *P. syringae* group that belong to the *Pseudomonas* genus. This may be due to the presence of boric acid in the KBC medium, toxic for many bacteria including some *Pseudomonas* species, and to the presence of trisodium citrate in the CVP medium that could be used as a carbon source in addition to the polypectate metabolized by SRP bacteria. Therefore, 16S metabarcoding analysis, by revealing the nature of the selective pressure exerted by a given medium, could in turn help to modify the medium to improve its selectivity. This point is particularly interesting when detection of rare pathogenic bacteria is needed.

In conclusion, 16S metabarcoding analyses proved a powerful tool to analyze and compare culture media selectivity. It is less time consuming than a classical culturomic experiment, where bacteria need to be isolated prior to characterization [3], and it could be directly performed on bacterial lawns where colonies are not well isolated. 16S metabarcoding also allowed a straightforward comparison of culture-grown communities with those from the initial environmental sample and more importantly, it allowed rapid detection of rare culturable species. Therefore, combining 16S metabarcoding analysis of environmental samples and culture-grown samples appeared powerful to accurately describe environmental samples without neglecting rare species of interest.

## Figures and Tables

**Table 1:** Average values of physicochemical and biological parameters at the sampling sites.

		Water Characterization				Estimated CFUs/Plate						
Site	Alt. m	Temp. °C	Conductivity µS	DOC mg C/L	Turbidity NTU	TSA 10% CFUs/Plate	KBC CFUs/Plate	PCR Estimated Psy/KBC Plate	% Estimated Psy/tot CFUs KBC	CVP CFUs/Plate	Visual Detection of CFUs SRP ^$^/Plate	% CFUs SRP ^$^/tot CFUs CVP
**UD**	790	10.5	354	0.93	85.8	2.20 × 10^5^	9.47 × 10^2^	100	9.4	1.38 × 10^3^	0	0
**MD**	620	11.4	609	0.67	3.1	4.36 × 10^5^	8.88 × 10^2^	50	5.8	1.91 × 10^3^	0	0
**LD**	188	15.2	511	1.03	31.8	3.40 × 10^5^	5.75 × 10^3^	13	0.2	3.55 × 10^3^	17	0.48

CFUs: colony-forming units; DOC: dissolved organic carbon; NTU: normalized turbidity unit; TSA 10%, KBC, CVP: culture media; Psy: *P.syringae*, SRP: soft rot *Pectobacteriaceae*.

**Table 2:** Sequencing data, α-diversity, and species evenness.

	Direct 16S Sequencing			16S Sequencing after Culture on								
Culture Medium	-			TSA 10%			CVP			KBC		
Site	UD	MD	LD	UD	MD	LD	UD	MD	LD	UD	MD	LD
**Total reads**	85,599 ± 2938	101,035 ± 3075	84,069 ± 11854	90,608 ± 10333	78,434 ± 8679	98,430 ± 12130	76,154 ± 7470	81,785 ± 9358	72,327 ± 13572	99,951 ± 3342	71,423 ± 5633	94,730 ± 1673
**Assigned reads**	60,248 ± 3516	73,796 ± 1735	54,233 ± 7161	48,712 ± 2715	39,252 ± 3584	52,778 ± 3506	41,229 ± 6368	45,519 ± 4981	42,132 ± 6664	54,372 ± 1357	37,808 ± 4772	50,880 ± 4019
**Good’s coverage**	0.994 ± 2.10^−4^	0.996 ± 9.10^−4^	0.994 ± 0.00	0.999 ± 1.10^−4^	0.999 ± 1.10^−4^	0.999 ± 1.10^−4^	0.999 ± 6.10^−5^	0.999 ± 1.10^−4^	0.999 ± 7.10^−5^	0.999 ± 1.10^−4^	0.999 ± 7.10^−5^	0.999 ± 2.10^−4^
**OTUs**	3793 ± 313	2142 ± 132	1269 ± 143	736 ± 108	699 ± 38	640 ± 71	392 ± 10	333 ± 49	349 ± 37	444 ± 49	281 ± 48	407 ± 30
**Shannon H Index**	6.76 ± 0.05	5.47 ± 0.18	3.93 ± 0.10	3.16 ± 0.24	3.20 ± 0.14	2.61 ± 0.06	1.74 ± 0.30	1.78 ± 0.17	1.80 ± 0.26	2.33 ± 0.16	2.09 ± 0.08	2.09 ± 0.26
**Pielou J Index**	0.82 ± 0.00	0.71 ± 0.03	0.55 ± 0.01	0.48 ± 0.04	0.49 ± 0.02	0.40 ± 0.01	0.29 ± 0.05	0.31 ± 0.02	0.31 ± 0.04	0.38 ± 0.03	0.37 ± 0.02	0.35 ± 0.04
**Phyla**	42 ± 1	37 ± 1	29 ± 1	6 ± 1	5 ± 0	5 ± 0	4 ± 1	5 ± 0	5 ± 0	5 ± 0	5 ± 1	5 ± 0
**Genera**	736 ± 13	540 ± 26	339 ± 10	120 ± 3	90 ± 8	97 ± 3	47 ± 2	48 ± 3	48 ± 4	49 ± 2	41 ± 5	44 ± 3

Mean and standard deviation (three replicates) are indicated. TSA 10%, KBC, CVP: culture media.

**Table 3:** Culture detection of abundant environmental OTUs: focus on the *Pseudomonas* genus.

	Site	*Pseudomonas*		
		*Pseudomonas* Reads	% Total Reads	Enrichment Factor Medium:Environment
**ENV**	**UD**	461	0.49	NA
	**MD**	454	0.49	NA
	**LD**	86	0.09	NA
**CVP**	**UD**	78719	84.41	171
	**MD**	78061	83.70	172
	**LD**	69772	74.81	811
**KBC**	**UD**	42406	45.47	92
	**MD**	54321	58.25	119.
	**LD**	27835	29.85	324
**TSA**	**UD**	7919	8.49	17
	**MD**	8716	9.35	19
	**LD**	5031	5.39	58

**Table 4:** Culture detection of rare OTUs: focus on the *Pectobacterium* genus.

	Site	*Pectobacterium*			
		CFUs SRP	% Total CFUs	SRP Reads	% Total Reads
**ENV**	**UD**	NA	NA	0	0
	**MD**	NA	NA	0	0
	**LD**	NA	NA	0	0
**CVP**	**UD**	0	0	16	0.017
	**MD**	0	0	2	0.002
	**LD**	51	0.48	928	0.995

## Data Availability

The datasets generated for this study are available in Table S1. Raw 16S sequence reads are available at the Sequence Read Archive (SRA: https://www.ncbi.nlm.nih.gov/sra) under the BioProject PRJNA645798.

## Supplementary Materials

The following are available online at https://www.mdpi.com/2076-2607/8/8/1129/s1, Figure S1: Optical Characteristics of Dissolved Organic Carbon (DOC). Figure S2: Rarefaction curves of the environmental Durance sample (ENV, blue) and cultivable samples (TSA: yellow, CVP: red, KBC: green). Table S1: Number of sequences observed for taxa assignations (line) for each treated sample (columns). Table S2: p-values of the student *t*-test between stations (Upper Durance, Medium Durance, and Lower Durance) for environmental and cultivable samples. Table S3: p-values of the student *t*-test between TSA/CVP/KBC cultivable samples at each station. Table S4: *p*-values of the student *t*-test between environmental and cultivable samples at each station Table S5: Analysis of specific/shared OTUs between cultured and environmental samples.
