# Supplementary material for: Comparison of Environmental and Culture-Derived Bacterial Communities through 16S Metabarcoding: A Powerful Tool to Assess Media Selectivity and Detect Rare Taxa
Source: Microorganisms. 2020 Jul 27;8(8):1129. doi: 10.3390/microorganisms8081129 (PMC7464939; doi:10.3390/microorganisms8081129)
Supplement: Supplementary file 1 [file microorganisms-08-01129-s001.zip › supplementals/table S2 .docx]

|  | **direct 16S sequencing** | | | | **16S sequencing after culture** | | | | | | | | | |
| --- | --- | --- | --- | --- | --- | --- | --- | --- | --- | --- | --- | --- | --- | --- |
| **culture medium** | **-** | | | | **TSA** | | | | **CVP** | | | **KBC** | | |
| **site** | **UD vs MD** | | **UD vs LD** | **MD vs LD** | **UD vs MD** | **UD vs LD** | **MD vs LD** | | **UD vs MD** | **UD vs LD** | **MD vs LD** | **UD vs MD** | **UD vs LD** | **MD vs LD** |
| **OTUs** | 0.005 | 0.001 | | 0.002 | 0.619 | 0.278 | 0.295 | 0.168 | | 0.178 | 0.672 | 0.015 | 0.343 | 0.025 |
| **Shannon H index** | 0.005 | | 0.000 | 0.001 | 0.784 | 0.052 | 0.008 | 0.882 | | 0.806 | 0.885 | 0.108 | 0.260 | 0.992 |
| **Pielou J index** | 0.023 | | 0.000 | 0.007 | 0.685 | 0.066 | 0.010 | 0.688 | | 0.691 | 0.938 | 0.647 | 0.300 | 0.437 |
| **Phyla** | 0.034 | | 0.005 | 0.000 | 0.225 | 0.225 | 1.000 | 0.184 | | 0.184 | 1.000 | 0.423 | 1.000 | 0.423 |
| **Genera** | 0.001 | | 0.000 | 0.002 | 0.014 | 0.000 | 0.241 | 0.393 | | 0.633 | 0.910 | 0.118 | 0.265 | 0.506 |

Table S2: p-values of student *t*-test between stations (Upper Durance, Medium Durance and Low Durance) for environmental and cultivable samples (independent two-sample Student *t*-test, unequal variance, two sided). Statistically different values are in red (p-values < 0.05).
