# Supplementary material for: Comparison of Environmental and Culture-Derived Bacterial Communities through 16S Metabarcoding: A Powerful Tool to Assess Media Selectivity and Detect Rare Taxa
Source: Microorganisms. 2020 Jul 27;8(8):1129. doi: 10.3390/microorganisms8081129 (PMC7464939; doi:10.3390/microorganisms8081129)
Supplement: Supplementary file 1 [file microorganisms-08-01129-s001.zip › supplementals/table S3.docx]

|  | **TSA vs CVP** | | | **TSA vs KBC** | | | **CVP vs KBC** | | |
| --- | --- | --- | --- | --- | --- | --- | --- | --- | --- |
| **site** | **UD** | **MD** | **LD** | **UD** | **MD** | **LD** | **UD** | **MD** | **LD** |
| **OTUs** | 0.031 | 0.001 | 0.008 | 0.031 | 0.001 | 0.008 | 0.206 | 0.261 | 0.108 |
| **Shannon H index** | 0.004 | 0.000 | 0.027 | 0.004 | 0.000 | 0.027 | 0.058 | 0.070 | 0.253 |
| **Pielou J index** | 0.008 | 0.001 | 0.062 | 0.008 | 0.001 | 0.062 | 0.065 | 0.028 | 0.323 |
| **Phyla** | 0.013 | 1.000 | 1.000 | 0.057 | 0.423 | 1.000 | 0.184 | 0.423 | 1.000 |
| **Genera** | 0.000 | 0.002 | 0.001 | 0.000 | 0.002 | 0.001 | 0.257 | 0.127 | 0.376 |

Table S3: p-values of student *t*-test between TSA / CVP / KBC cultivable samples at each station (independent two-sample Student *t*-test, unequal variance, two sided). Statistically different values are indicated in red (p-values < 0.05).
