# Supplementary material for: Comparison of Environmental and Culture-Derived Bacterial Communities through 16S Metabarcoding: A Powerful Tool to Assess Media Selectivity and Detect Rare Taxa
Source: Microorganisms. 2020 Jul 27;8(8):1129. doi: 10.3390/microorganisms8081129 (PMC7464939; doi:10.3390/microorganisms8081129)
Supplement: Supplementary file 1 [file microorganisms-08-01129-s001.zip › supplementals/table S4.docx]

|  | **ENV vs TSA** | | | **ENV vs CVP** | | | **ENV vs KBC** | | |
| --- | --- | --- | --- | --- | --- | --- | --- | --- | --- |
| **site** | **UD** | **MD** | **LD** | **UD** | **MD** | **LD** | **UD** | **MD** | **LD** |
| **OTUs** | 0.001 | 0.001 | 0.007 | 0.003 | 0.001 | 0.005 | 0.002 | 0.001 | 0.007 |
| **Shannon H index** | 0.001 | 0.000 | 0.000 | 0.001 | 0.000 | 0.002 | 0.000 | 0.000 | 0.003 |
| **Pielou J index** | 0.004 | 0.001 | 0.000 | 0.003 | 0.000 | 0.010 | 0.002 | 0.000 | 0.010 |
| **Phyla** | 0.001 | 0.000 | 0.000 | 0.000 | 0.000 | 0.000 | 0.001 | 0.000 | 0.000 |
| **Genera** | 0.000 | 0.000 | 0.000 | 0.000 | 0.001 | 0.000 | 0.000 | 0.001 | 0.000 |

Table S4: p-values of student *t*-test between environmental and cultivable samples at each station (independent two-sample Student *t*-test, unequal variance, two sided). Statistically different values in red (p-values < 0.05).
