# Supplementary material for: Comparison of Environmental and Culture-Derived Bacterial Communities through 16S Metabarcoding: A Powerful Tool to Assess Media Selectivity and Detect Rare Taxa
Source: Microorganisms. 2020 Jul 27;8(8):1129. doi: 10.3390/microorganisms8081129 (PMC7464939; doi:10.3390/microorganisms8081129)
Supplement: Supplementary file 1 [file microorganisms-08-01129-s001.zip › supplementals/Table S5.docx]

| **sample** | **site** | **specific OTUs** | **shared OTUs** |
| --- | --- | --- | --- |
| **ENV** | UD | 658.7 ± 8.4 | 77.3 ± 5.9 |
|  | MD | 477.0 ± 26.3 | 63.0 ± 4.4 |
|  | LD | 296.0 ± 8.9 | 43.0 ± 3.5 |
| **TSA** | UD | 46.3 ± 6.0 | 74.0 ± 5.2 |
|  | MD | 28.3 ± 4.0 | 61.7 ± 4.5 |
|  | LD | 56.3 ± 1.5 | 41.0 ± 4.0 |
| **CVP** | UD | 15.7 ± 1.0 | 31.0 ± 1.5 |
|  | MD | 15.3 ± 2.0 | 33.0 ± 0.6 |
|  | LD | 26.0 ± 2.6 | 22.0 ± 1.7 |
| **KBC** | UD | 15.7 ± 0.6 | 33.0 ± 1.7 |
|  | MD | 8.7 ± 2.3 | 32.7 ± 3.8 |
|  | LD | 10.3 ± 4.0 | 34.0 ± 1.0 |

**Table S5: Analysis of specific / shared OTUs between cultured and environmental samples:** for environmental samples (ENV), values represent specific and shared OTUs between environmental and all cultured samples. For cultured samples (TSA, CVP and KBC), values represent specific and shared OTUs between each cultured medium and environmental samples. Mean and standard deviation (3 replicates) are indicated. UD: upper Durance, MD: middle Durance, LD: lower Durance.
