# Supplementary figures and images for: Comparison of Environmental and Culture-Derived Bacterial Communities through 16S Metabarcoding: A Powerful Tool to Assess Media Selectivity and Detect Rare Taxa
Source: Microorganisms. 2020 Jul 27;8(8):1129. doi: 10.3390/microorganisms8081129 (PMC7464939; doi:10.3390/microorganisms8081129)

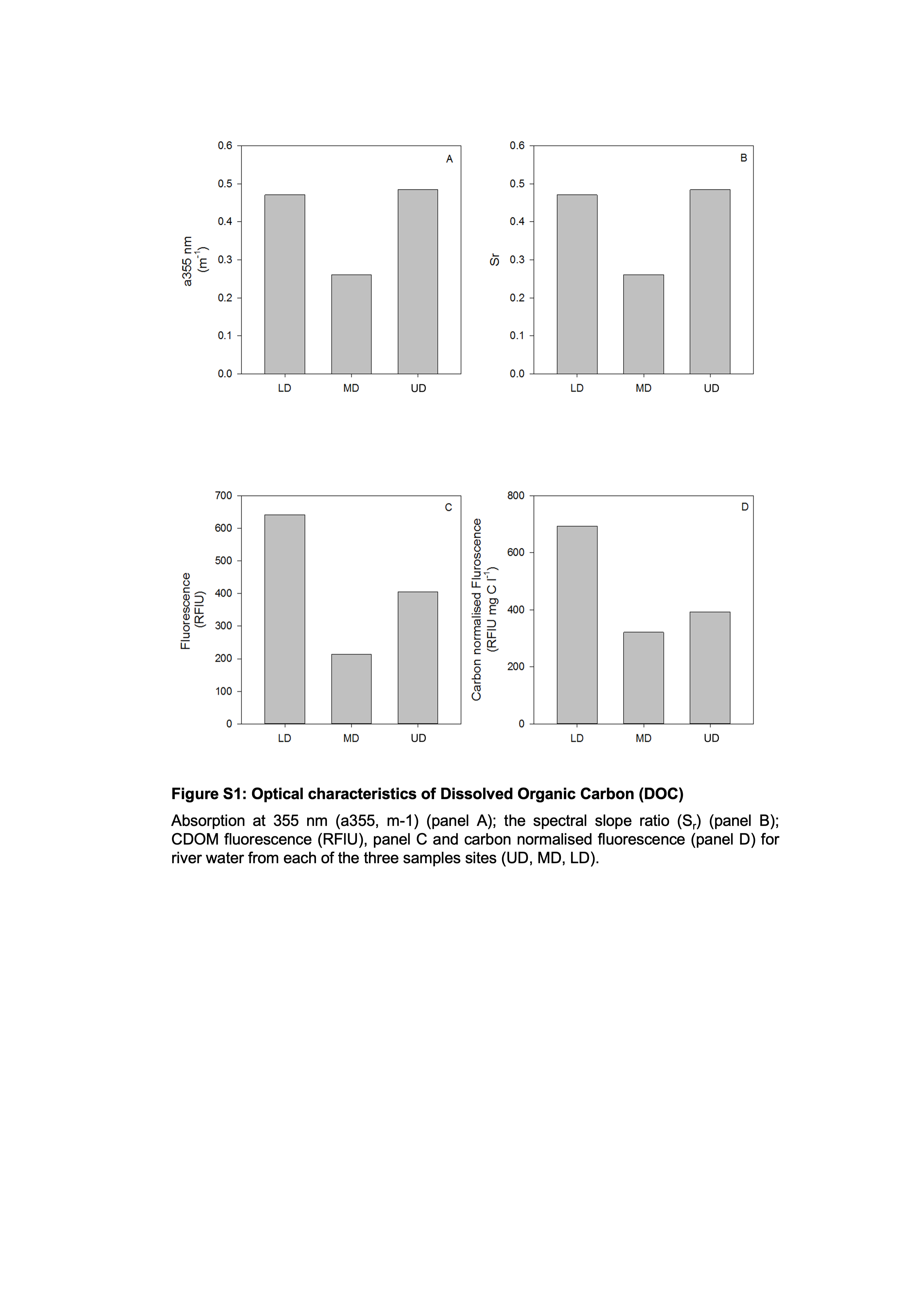

Supplement: Supplementary file 1 [file microorganisms-08-01129-s001.zip › supplementals/Figure S1-Pe╠üdron et al .tif]

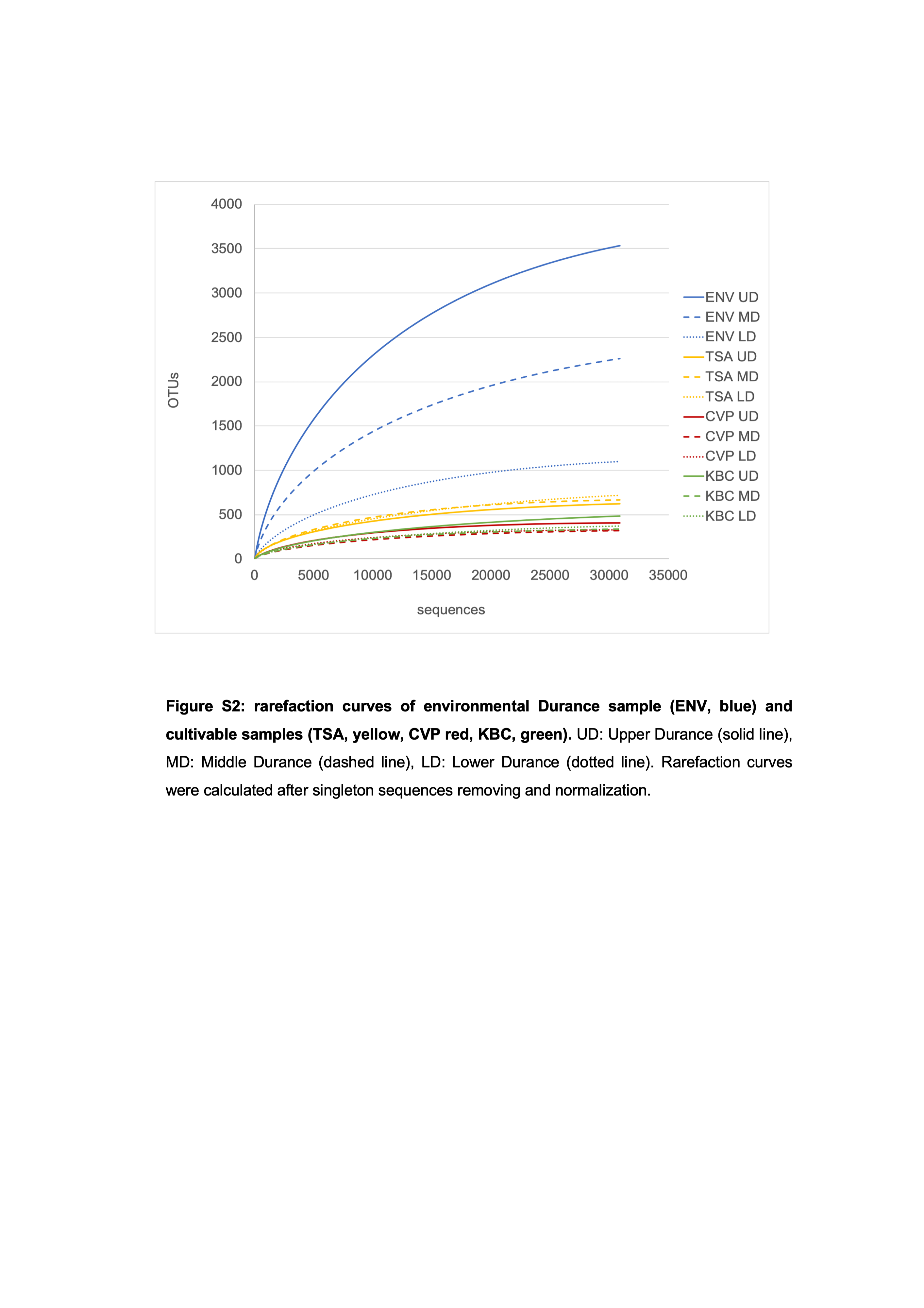

Supplement: Supplementary file 1 [file microorganisms-08-01129-s001.zip › supplementals/Figure S2-Pe╠üdron et al .tif]
